# Jaborandi as a Galactophore

**Published:** 1888-02

**Authors:** 


### ELECTROLYSIS IN THE TREATMENT OF OBSTRUCTION OF THE CANALICULUS.

The *British Medical Journal*, December 24, 1887, presents a report from Dr. W. E. Steavenson and W. H. Jessop, M. B., on the relief of epiphora, due to obstruction of the canaliculus, by the use of electricity· The number of cases reported is ten. They were consecutive and all of them relieved, at any rate for the present. In several instances the report would justify the assumption that it was drawn up by one not very familar with electrical observations; for example, we read "at the positive pole the metal is dissolved as well as the tissues." The expression may be justifiable if the electrode were made of the easily oxidizable metals, but would any one think of using any but a platinum electrode for such work. We might refer to several other expressions of a like character, but reserve the space for the main points of the operation.

Observations were confined to obstruction of the punctum and canaliculus: number of cases, ten; average age of patients, 38 years. Duration of affliction previous to treatment varied from ten weeks to seventeen years. The length of time the patient was under observation seems to average about three months, although the trouble seems to have been relieved frequently after two or three applications of the electricity. In two out of the ten cases, twenty per cent., a mistake was made in using the positive instead of the negative electrode in the canaliculus. The operation was confined in all the cases to the lower canaliculus.

The battery used consisted of four to six Stohrer's cells but it is deemed necessary to use a galvonometer on account of the difference which sometimes exists in the resistance of the tissues. Two to four milliamperes are said to be sufficient. The current is continued for a time varying from fifteen seconds to a minute ; the size of probe used to pass through the canaliculus varies from 0.5 mm. to 1.5 mm. The negative pole—never the positive—must be introduced through the canaliculus into the sac ; the positive can be applied on the nape of the neck. It is said that when a probe 0.5 mm. was inserted with difficulty immediately after the operation one of 1.00 mm. could be introduced easily. The probe should be attached to a handle furnished with a current-breaker, and care must be taken not to allow the current to pass directly through the eye. One of the two cases in which the positive pole was used in the canaliculus developed a lachrymal abcess, and it was found necessary to probe the other on alternate days so as to prevent occlusion ; both, however, are reported cured and the epiphora relieved.

### BOOK-REVIEWS.

Clinical Manual for the Study of Medical Cases. Edited by James Finlayson, M. D., Physician and Lecturer on Clinical Medicine in the Glasgow Western Infirmary; Physician to the Glasgow Hospital for Sick Children; President of the Glasgow Pathological and Clinical Society; Honorary Librarian to the Faculty of Physicians and Surgeons, Glasgow, etc. Second Edition. Revised and enlarged. With 158 illustrations. Philadelphia: Lea Brothers & Co., publishers. 1886.

The second edition of Professor Finlayson's most excellent Manual of Clinical Medicine is a very welcome addition to the very few good books on that subject. The work is too well known to require a description here. It is sufficient to say that the subject is thoroughly revised to the present date, and that every physician and student will find it a most pleasant and profitable companion.

F. B.

Hydrophobia. An Account of M. Pasteur's System. Containing a translation of all his communications on the subject, the technique of his method, and the latest statistical results. With seven illustrations. By Renaud Suzor, M. B. C. M., Edin., and M. D., Paris. London: Chatto & Windus, Picadilly. 1887. For sale by J. B. Lippincott Company, publishers, booksellers, and stationers, Philadelphia.

This small manual of 231 pages is divided into three parts. The first chapter, of 30 pages, gives a short description of hydrophobia, and its history from the earliest times down to the end of 1880.

The second chapter, of 128 pages, contains all the communications of M. Pasteur made to the Paris Academy of Medicine, with histories of cases treated.

The third chapter, of 83 pages, contains the technique of M. Pasteur's protective and curative method, with results of other operations, and a synopsis of a report of the British Parliamentary Commission, appointed to investigate Pasteur's method.

It is a valuable book to one who desires to know M. Pasteur's theories and methods of work.

The publishers' work is well done; the paper good, and the type clear. F. B.

Practical Microscopy. A Course of Normal Histology for Students and Practitioners of Medicine. By Maurice N. Miller, M. D., Director of the Department of Normal Histology in the Loomis Laboratory, University of the City of New York. Illustrated with one hundred and twenty-six photographic reproductions of the author's pen-drawings. Pp. 217. New York: William Wood & Company. Chicago: W. T. Keener.

The author's aim has been to guide the student in his technical work, and to give him sufficient instruction in the microscopic anatomy to enable him to correctly interpret what he sees. He gives the methods taught to his students in the Loomis Laboratory; these embody the simpler and most generally satisfactory processes of hardening, cutting, staining, and mounting tissues. And in the chapters devoted to the different tissues and organs, the methods best adapted to each are specially considered.

By far the greater part of the book is devoted to descriptive histology, illustrated by numerous cuts. The descriptions are condensed and not always lucid. The cuts are nearly all coarse; with few exceptions they are diagramatic, and in some instances misleading. This seems inexcusable in an age when book-illustration has become a fine art. The work does not reflect much credit upon either the author or the publishers.

Diseases of the Lungs and Pleuræ, Including Consumption. By R. Douglas Powell, M. D., Lond., Fellow of the Royal College of Physicians; Physician to the Middlesex Hospital, and to the Hospital for Consumption and diseases of the Chest, at Brompton; late Assistant Physician and Lecturer on Materia Medica at the Charing Cross Hospital. Third Edition. Re-written and enlarged, with illustrations, including two lithographic plates, being Vol. XI, of Wood's Library for 1886. New York: William Wood & Company. Chicago: W. T Keener.

The third edition of Professor Powell's book is an amplified edition of the work on "Consumption and on Certain Diseases of the Lungs and Pleuræ," which was published in 1878. The entire work of the old volume has been reconsidered and rewritten, and new chapters have been added on the Physical Examination of the Chest; on Asthma; on the Etiology of Phthisis; on the Complications of Phthisis; on the Surgical Treatment of Pulmonary Cavities; on Hydatid of the Lungs, and on Mediastinal Tumors.

An unusually large and satisfactory part of the book is devoted to the treatment of the diseases of the lungs and pleuræ. One chapter is devoted to the climatic treatment of phthisis, which will not be satisfactory to American physicians because our climate is not considered. The principles of climatic treatment indicated will enable one to select a suitable climate in our own country.

The chapters on pathology are very satisfactory, and the conclusions are in accord with those of the best pathologists of the age.

The publishers' work is very well done; the type is clear and easily read. F. B.

FEVER NURSING. Designed for the use of Professional and Other Nurses, and Especially for Nurses in Training. By J. C. WILSON, A. M., M. D. Pp. viii and 210. Philadelphia: J. B. Lippincott Company. 1888. Chicago: W. T. Keener.

This volume concludes the series of "Practical Lessons in Nursing," issued by its publishers. It "embodies the substance of a course of lectures on Fever Nursing, twice delivered before the Nurse-Class at the Philadelphia Hospital." This will prove the most generally useful of the series, and is an excellent book in every way.

The arrangement of topics is good, and the treatment of the points discussed is plain, concise, and as complete as the object intended demands. The literary style is clear and attractive. The book will be most useful as a text-book for nurses in training, and for this purpose it may be very highly commended. E. W.

DIET IN RELATION TO AGE AND ACTIVITY. By SIR H. THOMPSON, F. R. C. S. Pp. vi and 94. Boston: Cupples & Hurd. 1888. Chicago: W. T. Keener.

This is a re-publication, with some slight additions, of an article which first appeared in the *Nineteenth Century*. The author's reasons for the re-publication are "numerous demands," and "a profound conviction of the importance of the subject." He states it as his conviction that "more than one-half of the disease which embitters the middle and latter part of life among the middle and upper classes of the population is due to avoidable errors in diet."

The book consists mainly of a dignified and graceful protest against the idea so prevalent among the classes mentioned, that an increase in weight is desirable as old age approaches. E. W.

DOCTOR AND PATIENT. By S. WEIR MITCHELL, M. D., LL. D. Pp. 177; 12 mo. Philadelphia: J. B. Lippincott Company. 1888. Chicago: W. T. Keener.

The very high esteem in which the author of this book is held, both as a writer and as a physician, is amply sustained by the merits of this his latest volume. It consists in a number of essays which deal with the mutual relation of physician and patient, and are intended to interest the laity more than the medical attendant, and are particularly addressed to nervous women. The book will find its largest circle of readers among the laity, and they will derive the most practical benefit from it, but its most appreciative readers will be those doctors who realize that the richest rewards which their work brings them are not financial ones. The author says that he was tempted when he wrote these essays to call them lay sermons, so serious did some of their subjects seem to him. "They touch, indeed, on matters involving certain of the most difficult problems in human life, and involve so much that goes to mar or make character, that no man could too gravely approach such a task."

A list of the titles of the essays can convey a better idea of the scope of the volume than anything which the limits of such a notice as this allows, and is consequently given here: Introductory; The Physician; Convalescence; Pain and its Consequences; The Moral Management of Sick or Invalid Children; Nervousness and its Influence on Character: Out-Door and Camp-Life for Women.

The fact that the value of a book bears no sort of relation to its size is strikingly exemplified in the volume under consideration. The overworked doctor, who is at times tempted to think that too much bread which never returns is cast by him upon the waters of an unappreciative clientéle, will find a wealth of comfort and solace in reading its suggestive and gracefully written pages.

LECTURES ON THE DIAGNOSIS OF DISEASES OF THE BRAIN. By W. R. GOWERS, M. D., F. R. S. Second Edition, pp. vii, 254. Philadelphia: P. Blakiston, Son & Company. Chicago: W. T. Keener.

This second edition of Gowers' eighteen lectures is issued by the Messrs. Blakiston & Company in the neat dress which characterizes the books which come from that well-known house. It is free from that affectation of uncut leaves of English publishers generally, which necessitates the trouble of cutting the leaves, and which causes an unsightly edge as a consequence.

The author has thoroughly revised the book and corrected some defects which existed in the first edition.

In the opening lecture, as an appropriate introduction, the "Medical Anatomy of the Brain" is given briefly, preparatory to considering its normal action. Then, as the book is designed as an aid to diagnosis, the abnormal conditions, whether functional or organic, are presented in so clear and interesting a manner as to make it one of the best manuals on that subject in the English language. The lectures which treat of impairment of the function of the special senses are particularly interesting.

TEXT-BOOK OF THERAPEUTICS AND MATERIA MEDICA. Intended for the Use of Students and Practitioners. By ROBERT T. EDES, A. B., M. D., Professor of Materia Medica and Jackson Professor of Clinical Medicine in Harvard University, etc. Pp. xi, 552. Philadelphia: Lea Brothers & Co. Chicago : A. C. McClurg & Co.

This is an elementary text-book avowedly written, compiled, and abstracted for the benefit of "overburdened students and young practitioners." Doubtless there is a legitimate demand for the best of such books, of which this is certainly one, since with the advance of medical science the less useful information must be eliminated in order that the student may learn the more truly valuable. It would seem, however, that the pruning process might have been less vigorously applied to the parts pertaining to pharmacology. The physiological action of digitalis, for instance, is treated in a manner wholly incommensurate to its importance, and the physiological effects of belladonna as represented by its alkaloidal salt, sulphate of atropia, are tabulated with a brevity which renders them difficult of comprehension except to those already familiar with the subject. Many details of materia medica proper and pharmaceutical data can well be left to the educated pharmacist, but medical students should be taught with thoroughness and precision modern pharmacology and the associated therapeutic principles which underlie the rational application of drugs to the cure of disease.

The work contains many excellent features. The classification, in part original, is well devised, and includes within its scope all the newer remedies. Among hypnotics we note paraldehyde, urethran, and hypnone ; among antipyretics, salol, antipyrine and antifebrine ; among cardiac stimulants, strophanthus ; while iodol, resorcin, lanolin, "cucaina" (cocaine), and others, receive due consideration. Useful alkaloids and active principles, also, are noticed at length.

The typographical arrangement is such that the names of the more important drugs and preparations appear in "Roman Capitals," the less important in "small capitals," and the non-officinal inclosed within brackets.

The phraseology is clear and concise, and at the same time colloquial in style, so that the book is not by any means the "dry reading" which its subject is ordinarily presumed to supply. It can be perused with pleasure and profit by all. W. E. C.

